# Stage-resolved transcriptomic profiling of *Anastrepha ludens* (Diptera: Tephritidae) from egg to adult: molecular signatures of a notorious polyphagous fruit-fly pest

**DOI:** 10.3389/finsc.2025.1618382

**Published:** 2025-08-29

**Authors:** Daniel Cerqueda-García, Ixchel Osorio-Paz, Javier Carpinteyro-Ponce, Enrique Ibarra-Laclette, Alma Altúzar-Molina, Martín Aluja

**Affiliations:** ^1^^®^ Red de Manejo Biorracional de Plagas y Vectores, Instituto de Ecología, A.C. - INECOL, Clúster Científico y Tecnológico Biomimic^®^ , Xalapa, Veracruz, Mexico; ^2^ Biosphere Sciences and Engineering Division, Carnegie Institution for Science, Baltimore, MD, United States; ^3^^®^ Red de Estudios Moleculares Avanzados, Instituto de Ecología, A.C. – INECOL, Clúster Científico y Tecnológico Biomimic^®^ , Xalapa, Veracruz, Mexico

**Keywords:** *Anastrepha ludens* (Diptera: Tephritidae), transcriptional dynamics, transcriptomic profiling, gene expression patterns, metabolic pathways

## Abstract

This study explores the transcriptional dynamics of the polyphagous Mexican Fruit Fly, *Anastrepha ludens*, across five developmental stages, revealing distinct gene expression patterns unique to each stage. We identified 9,762 DEGs associated with the four developmental stages. During the egg stage, we identified the greatest number of differentially expressed genes exhibiting a pronounced activity of metabolic pathways, particularly the Mitogen-Associated Protein Kinase (MAPK) signaling pathway, which is essential for embryonic development and defense mechanisms. The second larval instar stage mainly focused on growth, as shown by the overexpression of the Transforming Growth Factor Beta (TGF-beta) pathway. In the third larval stage, genes are significantly enriched in cuticle structure and transmembrane transport. In the pupal stage, the importance of the TGF-beta and mTOR pathways emerged, vital for tissue homeostasis and development. The adult stage exhibited sustained expression of the FOXO pathway, enhancing stress resistance crucial for survival and reproduction. Additionally, we noted differences in odor-binding protein (OBP) expression between sexes, hinting at their potential role in mating behavior. These findings provide fundamental information about the life stages of *A. ludens*, highlighting the importance of specific signaling pathways and OBPs, which could help improve mass rearing processes and management strategies for this notorious tephritid pest.

## 1 Introduction

The widespread adoption of transcriptomics has transformed our ability to chart the dynamic gene-expression programs of insect pests. Stage-resolved datasets now exist for *Tribolium castaneum* (Herbst) (Coleoptera: Tenebrionidae) (1), *Bactrocera dorsalis* (Hendel) (Diptera: Tephritidae) (2), *Anopheles gambiae* Giles (Diptera: Culicidae) (3), *Acheta domesticus* L. (Orthoptera: Gryllidae) (4) and *Bactrocera minax* Enderlein (Diptera: Tephritidae) (5), among others, and point to genes whose manipulation could underpin futuristic, molecularly based control strategies. Translating such targets between species, however, is not always straightforward: differences in gene function, RNA-interference efficiency, and even gut uptake of double-stranded RNA can limit direct transferability (e.g. 6, 7). At the same time, notable successes—such as the developmental-gene interventions reported for *Drosophila suzukii* (Matsumura) (Diptera: Drosophilidae) (8)—demonstrate the practical value of species-specific transcriptome resources. Our study therefore aims at extending this knowledge base by identifying stage-enriched genes in *Anastrepha ludens* (Loew) (Diptera: Tephritidae) that may support the development of biologically based, environmentally-friendly pest-management tools.

The highly polyphagous Mexican Fruit Fly, *A ludens*, is distributed from the southern United States to Panama (9, 10). Like other tephritid pests, female *A. ludens* lay batches of eggs (up to 40 eggs per batch) into a fruit (11), and larval feeding causes extensive pulp decay and premature fruit drop. This pestiferous fly species attacks numerous wild and cultivated plants, including *Casimiroa edulis*/*C. greggii* (wild/ancestral hosts), citrus (mainly grapefruit and *Citrus x aurantium*), mango, peaches, manzano pepper, and pomegranate (12, 13). Consequently, it is considered one of the most economically important fruit pests across its distribution range and a significant threat elsewhere due to global climate change (10, 14, 15). Therefore, there is an urgent need to gain further insight into this pest’s biology, ecology, genetics, and behavior using state-of-the-art omics tools.

Various methods have been developed to combat this pest, ranging from conventional control using bait sprays laced with insecticides such as Malathion and more recently Spinosad^®^ (a bacterially derived insecticide) (16–18) to the use of synthetic host marking pheromones (19), and transgenic genetic sexing for enhancement of Sterile-Insect-Technique (SIT)-based programs (18). SIT involves the mass production and release of sterile males and can be more cost-effective and environmentally friendly than conventional control methods (20). Sterilization can be achieved through irradiation (21–24) or using genetic manipulation of the insects. For example, a dosage-dependent Tet-off transgenic embryonic sexing system (TESS) has been developed, which induces female lethality during embryogenesis and results in 100% male progeny (25). A similar approach using CRISPR/Cas9 has been described in *Anastrepha suspensa* (Loew) (Diptera: Tephritidae), targeting the Transfomer-2 (tra2) gene involved in female development (26). However, not all methods relying on genetic manipulation focus on male sterility, as some are based on a conditional lethal system during the developmental stages (27). Furthermore, the CRISPR/Cas9 gene editing technique has shown promise in optimizing SIT by targeting sex-determination genes in insects such as *Drosophila melanogaster* (Diptera: Drosophilidae) and *A. gambiae*, to improve male fitness (28, 29).

Recent transcriptomic studies have shed light on the genetic underpinnings of critical aspects of the biology of pestiferous tephritid species. For instance, an in-depth transcriptomic analysis of the melon fly, *Zeugodacus cucurbitae* (Coquillett), (Diptera: Tephritidae) revealed notable effects on the development of larvae that stemmed from irradiated eggs, specifically implicating the ImpE2 and Tpk-tok genes. The suppression of these genes resulted in premature pupation, hindered larval development, and ultimately led to mortality (30). In a study on *B. dorsalis*, two genes encoding the receptors BdorOR13a and BdorOR7a-6 were identified in females. These olfactory receptors (ORs) react to the volatile compound 1-octen-3-ol and open promising avenues for developing effective female attractants for monitoring *B. dorsalis* (31). It has been hypothesized that ORs efficiently perceive odorants from the environment, perhaps by changing their three-dimensional structure and allowing their interaction with odorant-binding proteins (OBPs). The latter are present in high concentrations in the sensillar lymph and their early interaction with the odorants triggers the transduction cascade of olfactory signals (32, 33).

Recent stage-resolved and functional transcriptomes within *Anastrepha* are expanding the molecular toolkit: Segura-León et al. (34) characterized 120 chemosensory genes from adult head transcriptomes of *A. ludens*; Lemos-Lucumí et al., (35) produced the first third-instar larval micro-transcriptome of *Anastrepha obliqua (Macquart)*, linking gene-expression signatures to polyphagy; Cárdenas-Hernández et al. (36) demonstrated, via comparative larval metatranscriptomics, that gut-microbiota gene expression shifts with host fruit—knowledge that could facilitate probiotic or diet-based refinements of SIT; and Scannapieco et al. (37) assembled stage- and sex-specific transcriptomes for *Anastrepha fraterculus* Wiedemann (Diptera: Tephritidae), providing an additional resource for identifying developmental and reproductive gene targets. These studies underscore the significance of characterizing the transcriptomic profiles across different developmental stages of pests such as *A*. *ludens*, and offer valuable insights for more efficient and targeted pest control strategies.

In this study, we performed a comprehensive transcriptomic analysis of the life cycle of *A. ludens*, including the egg, two larval stages, pupae, and female and male adults. We aimed at gaining further insight into changes in gene expression during each life stage of this important fruit pest. This analysis builds upon and significantly expands the valuable information on the cytological and transcriptomic analyses of *A. ludens*’ embryonic development published by Gutiérrez-Ramos et al. (38). The morphology of the immature stages of *A. ludens* was redescribed by Carroll and Wharton (39), and we utilized this information to distinguish between the two larval stages studied. By integrating these findings with our transcriptomic analysis, we hope to significantly contribute toward a comprehensive understanding of the molecular processes and developmental events underlying the life cycle of *A. ludens*, shedding light on its biology and potential enhancements on mass rearing, monitoring, and management.

## 2 Materials and methods

### 2.1 Biological material

We obtained samples of all developmental stages of *A. ludens* from a laboratory colony established by the Red de Manejo Biorracional de Plagas y Vectores at the Clúster Científico y Tecnológico Biomimic^®^, Instituto de Ecología, A.C. – INECOL. The *A. ludens* colony has been kept under continuous rearing in the laboratory for ca. 130 generations but was refreshed with wild material from citrus fruit in 2015, following conditions described by Aluja et al. (40). We underline the fact that we decided to use a genetically more stabilized colony than a genetically more variable field population to compare the results of ongoing transcriptomic studies. Briefly, *A. ludens* adults were kept since their emergence in 30x30x60 cm plexiglass cages under controlled conditions (27 ± 1°C, 63 ± 5% of relative humidity, and a photoperiod of 12:12 h light: dark) and fed *ad libitum* with an artificial diet consisting of hydrolyzed protein: sucrose (1:3) and water. Three samples (*i.e*., replicates) per sex with eight 8-day-old individuals per sample were used for the transcriptomic analysis of this developmental stage.

To sample eggs, an oviposition device filled with furcellaran (TIC pretested^®^ Burtonite 44 C Powder, TIC Gums, Belcamp, MD, USA) gel (11 g/L) was placed on top of the roof of a cage containing ca. 7,000 sexually mature flies (mated females and males) during 24 h. Eggs were separated from the furcellaran by rinsing them with sterile water and excess of water was removed with a micropipette. In the end, 300 mg (fresh weight) of eggs (7,594 eggs on average) per replicate (total of three replicates) were used for the transcriptomic analysis.

Larvae reared at 29 ± 1°C, 70 ± 5% relative humidity, and dark conditions were fed *ad libitum* with an artificial diet containing corn cob powder (11.89%), yeast (7.93%), sugarcane (7.93%), wheat germ (7.93%), sodium benzoate (0.47%), citric acid (0.4%), and purified water (63.45%) (further details in 41). Pools of 40 and 10 individuals per replicate (we ran a total of three replicates) of five- and eight-day-old larvae, respectively, were used for transcriptomic analysis. The logic behind the 40/10 individuals per larval stage was to homogenize the amount/weight of tissue in both stages, as second instar larvae (five-day old) are significantly smaller than third instar larvae (eight-day old). A second instar larvae measures on average ± S.E. 9.71 ± 0.8 mm (N = 25), whereas a third instar larvae measures 11.09 ± 0.4 mm (N = 25), respectively. Finally, pupae were kept at 22 ± 1°C, 70 ± 5% relative humidity, and dark conditions. Samples of pupae consisted of pools with ten eight-day-old pupae per replicate (three replicates in total).

Samples of all developmental stages were frozen in liquid nitrogen and stored at -80°C until they were analyzed. Three biological replicates were sampled for each developmental condition.

It is important to note that all transcriptomes were generated using whole-body samples for each developmental stage. We acknowledge that while our approach provides a global view of gene expression, it limits the ability to resolve tissue-specific expression, particularly for genes such as those involved in circadian regulation, which may be expressed in specific neuronal or peripheral tissues.

### 2.2 RNA-seq sample processing, assembly, and annotation

To minimize circadian-related variability in gene expression, all samples for the different developmental stages were collected at the same circadian time point, specifically at Zeitgeber Time 6 (ZT6)—six hours after lights-on—under controlled 12:12 h light: dark (LD) conditions. This synchronized sampling approach was implemented uniformly across all life stages and biological replicates to ensure comparability of gene expression levels, particularly for genes known to be under circadian regulation.

For RNA extraction, we used 200 mg of homogenized tissue following the instructions of the Plant/Fungi RNA purification kit (NorGen Biotek Corp; Thorold, ON, Canada). The purity of RNA samples was determined by measuring absorbance at 230 and 280 nm. Samples were also analyzed using the Bioanalyzer 2100 (Agilent Technologies), and only RNAs with RIN values above 8.0 were used to generate the libraries for sequencing. RNA-seq library construction was performed at the High-Throughput Sequencing Unit of the Institute of Ecology, A.C. (INECOL). For each condition, 3.5 μg of RNA were used with the TruSeq™ RNA Sample Preparation v2 kit (Illumina). Library quality analysis was carried out using the Bioanalyzer 2100 capillary electrophoresis system (Agilent Technologies, Santa Clara, USA). Each cDNA library was normalized to a final concentration of 20 mM based on the results provided by the Bioanalyzer equipment. Finally, the libraries were divided into groups to be sequenced on the NextSeq500 equipment (Illumina) generating paired end reads in the 2 x 150 bp format. The libraries were collaboratively sequenced with the Advanced Genomics Unit (UGA-Langebio), CINVESTAV.

We processed the 18 RNA-seq libraries removing the low-quality reads and trimming barcodes using fastp with default parameters. Pre-processed samples were then fed into Trinity v2.15.0 for generating a *de novo* transcriptome assembly. All samples were read independently into Trinity using the –sample_file argument. Final transcriptome assembly was constructed based on all developmental stages. We then processed the resulting assembly with TransDecoder to obtain the longest isoforms and predict open reading frames (ORFs). We retained ORFs whose similarity scores to known proteins were significant as determined by BLASTP searches against UniProt. The resulting BLAST hits were supplied to TransDecoder.Predict with the –retain_blastp_hits flag, so that regions showing coding-like sequence features and high sequence similarity to UniProt entries were preferentially retained (Haas et al., https://github.com/TransDecoder/TransDecoder).

We annotated the predicted amino acid sequences with the InterProScan (42) and the eggNOG-mapper (v5.0) (43) pipelines. The GO identifiers produced by InterProScan were parsed with GSEABase (44) to retrieve their full-term names and ontology categories (BP, MF, CC), match each identifier to the *Drosophila* GO-slim subset, and build a GeneSetCollection object that links every unigene to its corresponding GO terms for downstream analyses.

### 2.3 Determination of differentially expressed genes throughout developmental stages

All libraries were mapped individually to a single global *de novo* transcriptome assembly constructed from pooled reads across all developmental stages. This strategy is widely recommended for studies in non-model organisms, as it increases unigene recovery and improves transcript contiguity and annotation (45–47).

We quantified the transcript expression for each library using Salmon (48) and exported the quantification files to the R environment using the tximportData library (49). Normalization factors were calculated in edgeR (50). Differential expression was then evaluated in limma-voom using a single quadratic time-course model: the five developmental stages (egg, larval instar 2, larval instar 3, pupa, adult) were coded as an ordered numeric variable and fitted with linear and quadratic terms (51). This approach tests each gene’s expression trajectory across the entire life cycle—rather than making separate pair-wise contrasts—and detects genes that rise, fall, or peak at the developmental stage at which each gene reached its maximum expression value. Unigenes were considered differentially expressed when the overall time-course model was significant (FDR < 0.01), independent of the direction of change. For every significant gene, we identified the developmental stage at which the fitted expression curve reached its maximum value and labeled the gene as stage-enriched at that point.

Lastly, using KEGG annotations, we assigned each stage-enriched DEG to its corresponding pathway and, for every developmental stage, compiled the pathways represented by the genes that reached their maximum expression at that stage.

## 3 Results

### 3.1 *De novo* transcriptome analysis

The transcriptomic libraries comprise seventeen datasets with a mean of 31.16 million high-quality reads (min: 23.24, max: 60.22). The BUSCO analysis determined that the assembly is highly complete, with 3,011 of the 3,114 expected orthologue groups being recovered (96.7%). Of these, 904 BUSCOs were present as single-copy genes, whereas 2,107 were detected in duplicated copies, suggesting either residual haplotype redundancy or genuine gene duplication. Only 56 BUSCOs were fragmented (1.8%), and 47 were entirely missing (1.5%). Overall, these metrics indicate a near-complete representation of the conserved gene set in the assembly, and independent mapping of each stage’s libraries showed that over 97% of these conserved genes had transcriptional evidence at each stage (see **Supplementary Table S1**).

**Figure 1:**
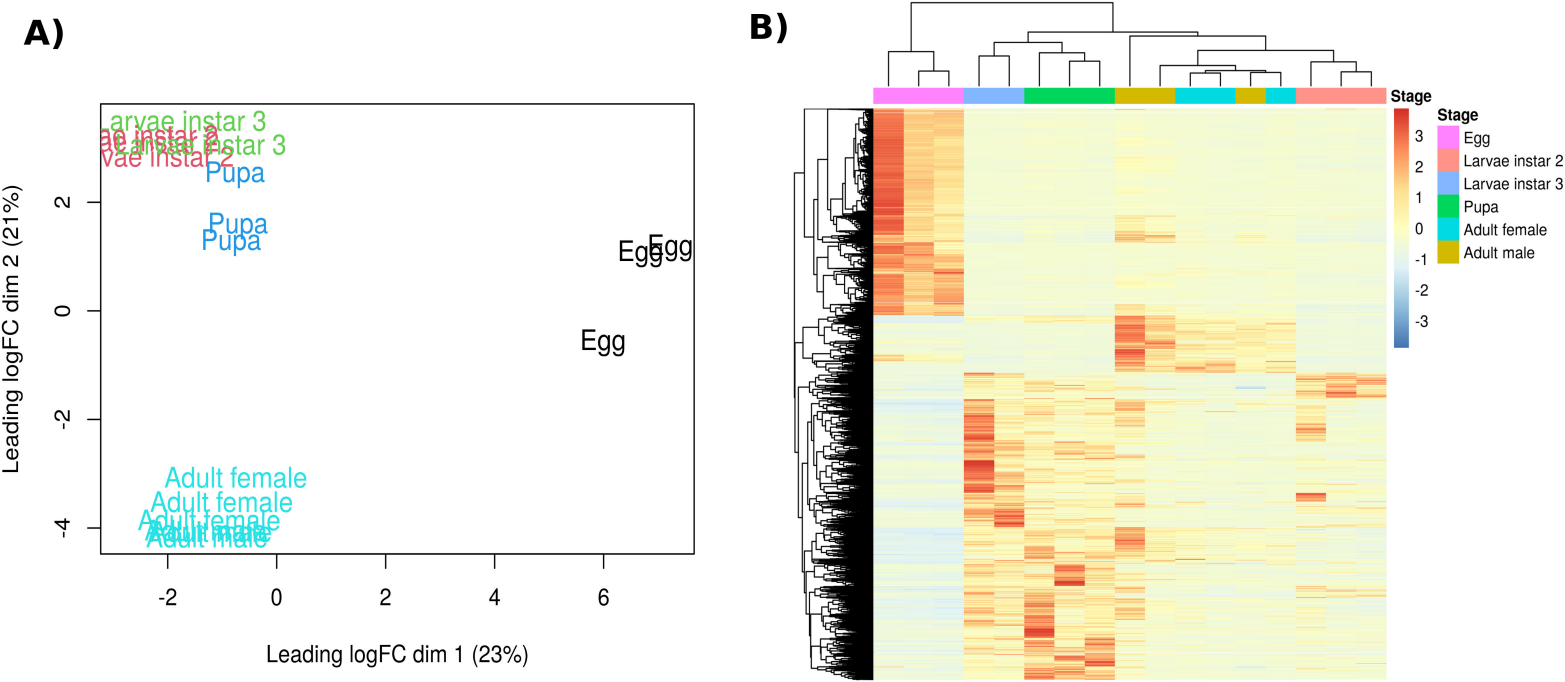
**(A)** Principal‐components analysis of log_2_-CPM expression values for all libraries (PC1 = 23% variance; PC2 = 21%). Biological replicates cluster by developmental stage (egg – black; L2 – red; L3 – green; pupa – blue; adult – cyan). **(B)** Heat map of the same samples showing hierarchical clustering of stage-enriched differentially expressed unigenes. Rows represent genes, columns represent samples ordered by stage, and the top color bar indicates stage identity. Color intensity reflects normalized expression (log_2_-CPM).

The assembly resulted in 66,968 longest transcript isoforms. A principal-components analysis of all transcripts (**Figure 1A**) separates libraries broadly by stage: eggs at positive PC1 values; pupae and third-instar larvae at intermediate PC2 positions; adult males and females toward negative PC2; and second-instar larvae near the origin. The heat-map in **Figure 1B**, generated from stage-enriched differentially expressed genes, clusters eggs, L2, L3, pupae, and adults into distinct groups, with L3 aligning with pupae and L2 aligning with adults.

### 3.2 Differentially expressed unigenes related to developmental stages

Quadratic linear regression identified 9,762 DEGs associated with the developmental stages. The stage with the highest gene expression (peaking) was the egg stage, with 3,439 DEGs. The two larval instars studied had 691 DEGs in stage two and 2,730 in stage three. The pupal stage had 1,710 DEGs at its peak, while adults had 483, and 709 DEGs for females and males, respectively, totaling 1,192. The sequences and DEGs annotations are available in the Supplementary Material.

### 3.3 Molecular functions related to different stages

**Figure 2:**
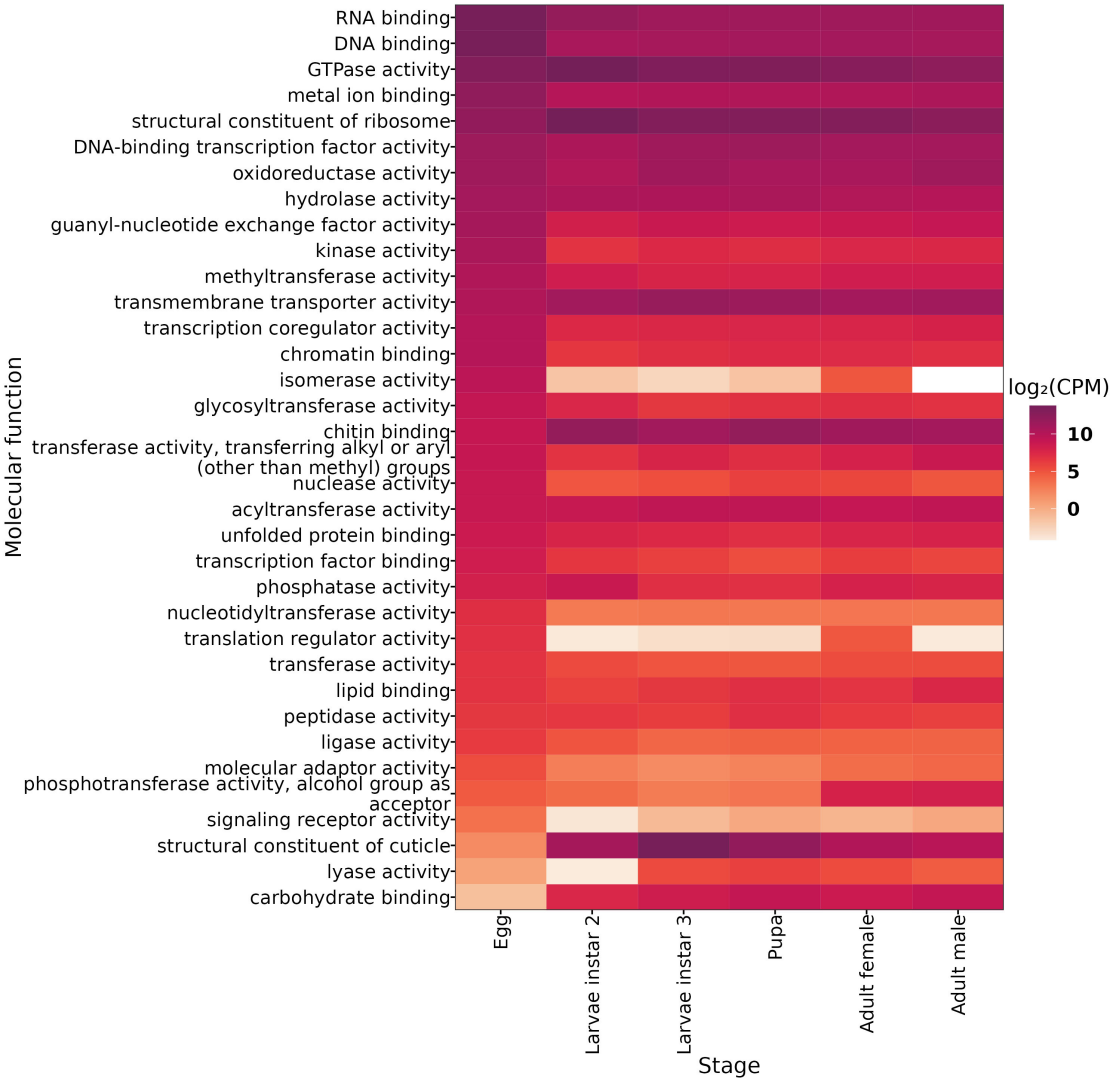
Differentially Expressed Genes (DEGs) for each developmental stage grouped in the molecular function of gen ontology. Expression values are shown in log_2_-CPM (counts per million).

Our results show that the egg stage is characterized by enrichment in 19 molecular functions, such as RNA binding, DNA binding, metal ion binding, and hydrolase activity. Unigenes involved in transcription regulation, chromatin binding, and protein modification are also highly enriched (**Figure 2**). During the larval stage, we found enrichment in three molecular functions in the second instar: the ribosomal structure and function, GTPase activity, and phosphatase activity. In larval stage 3, genes are significantly enriched in cuticle structure and transmembrane transport. In the pupal stage, we found enrichment in six molecular functions, including chitin binding, DNA-binding transcription factor activity, acyltransferase activity, carbohydrate binding, peptidase activity, and lyase activity. Finally, in adult male *A. ludens*, we found enrichment in three molecular functions: oxidoreductase activity, phosphotransferase activity with alcohol group as acceptor, and lipid binding.

### 3.4 Selected KEGG pathways related to developmental stages

**Figure 3:**
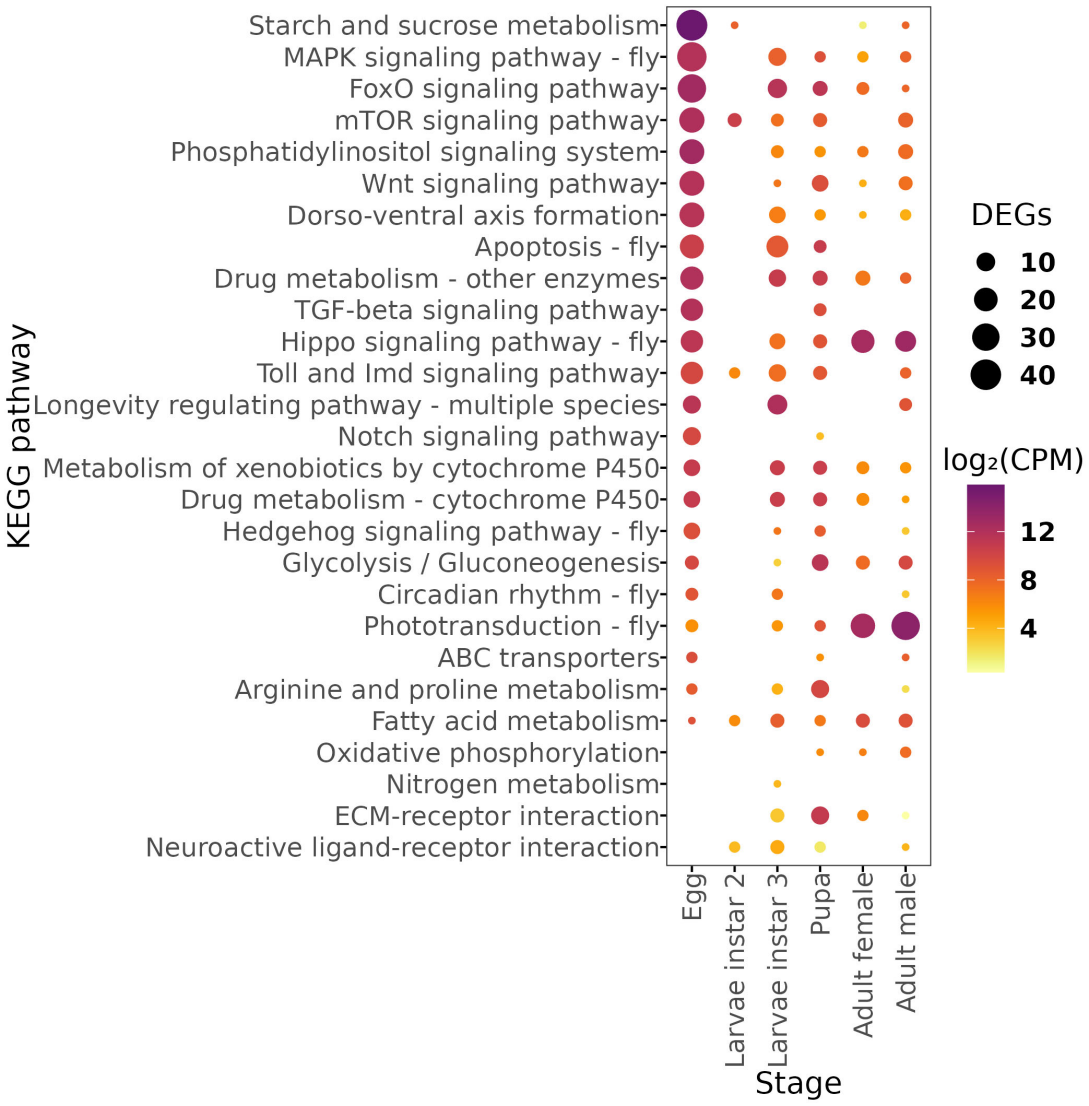
Dot plot of number of genes and abundance of Differentially Expressed Genes (DEGs) mapped in the KEGG metabolic pathways for each developmental stage. Expression values are shown in log_2_-CPM (counts per million).

Using the KEGG database, we identified the peaking pathways in the different developmental stages of *A. ludens* (**Figure 3**). Our analysis revealed that the Starch and sucrose metabolism pathway, FoxO signaling pathway, Phosphatidylinositol signaling system, mTOR signaling pathway, Drug metabolism - other enzymes, TGF-beta signaling pathway, MAPK signaling pathway - fly, Wnt signaling pathway, Dorso-ventral axis formation, Drug metabolism - cytochrome P450, Metabolism of xenobiotics by cytochrome P450, Toll and Imd signaling pathway, and Hedgehog signaling pathway - fly were significantly enriched in the egg samples of *A. ludens*. These pathways are involved in various cellular processes, including signal transduction, cell growth and differentiation, drug metabolism, and detoxification of xenobiotic compounds. These findings suggest that the embryos of *A. ludens* are actively modulating their metabolism and stress response pathways to support embryonic development. An intriguing result was the enrichment of Circadian rhythm – fly pathway in the egg, given that in other insects, such as *D. melanogaster*, a functional central circadian clock typically emerges during the larval instar 2. The early expression in eggs might reflect preparatory transcriptional events or peripheral circadian activity in non-neural tissues. However, since whole-body samples were used, gene expression from functionally relevant tissues may have been masked by other tissues with weak or no rhythmic activity. Therefore, these results must be interpreted with caution, and future studies incorporating tissue-specific transcriptomics are warranted.

Furthermore, our analysis showed that the longevity regulating pathway - multiple species, neuroactive ligand-receptor interaction, and nitrogen metabolism were significantly enriched in the third instar larvae of *A. ludens*. These pathways are involved in regulating neuronal signaling, protein metabolism, and nitrogen balance, respectively, suggesting their importance in larval development (52–54). In the pupal stage, we observed significant enrichment of the Glycolysis/Gluconeogenesis pathway, ECM-receptor interaction, Apoptosis - fly, Arginine and proline metabolism, and Fatty acid metabolism. These pathways are involved in energy metabolism, cell signaling, programmed cell death, and amino acid metabolism, suggesting their importance in the pupal development of *A. ludens* (55–58). In the adult stages, we observed significant enrichment of the Phototransduction - fly pathway in males (59) and the Hippo signaling pathway - fly and Oxidative phosphorylation pathways in females (60, 61).

### 3.5 Chemoreception and odorant-binding proteins throughout developmental stages

**Figure 4:**
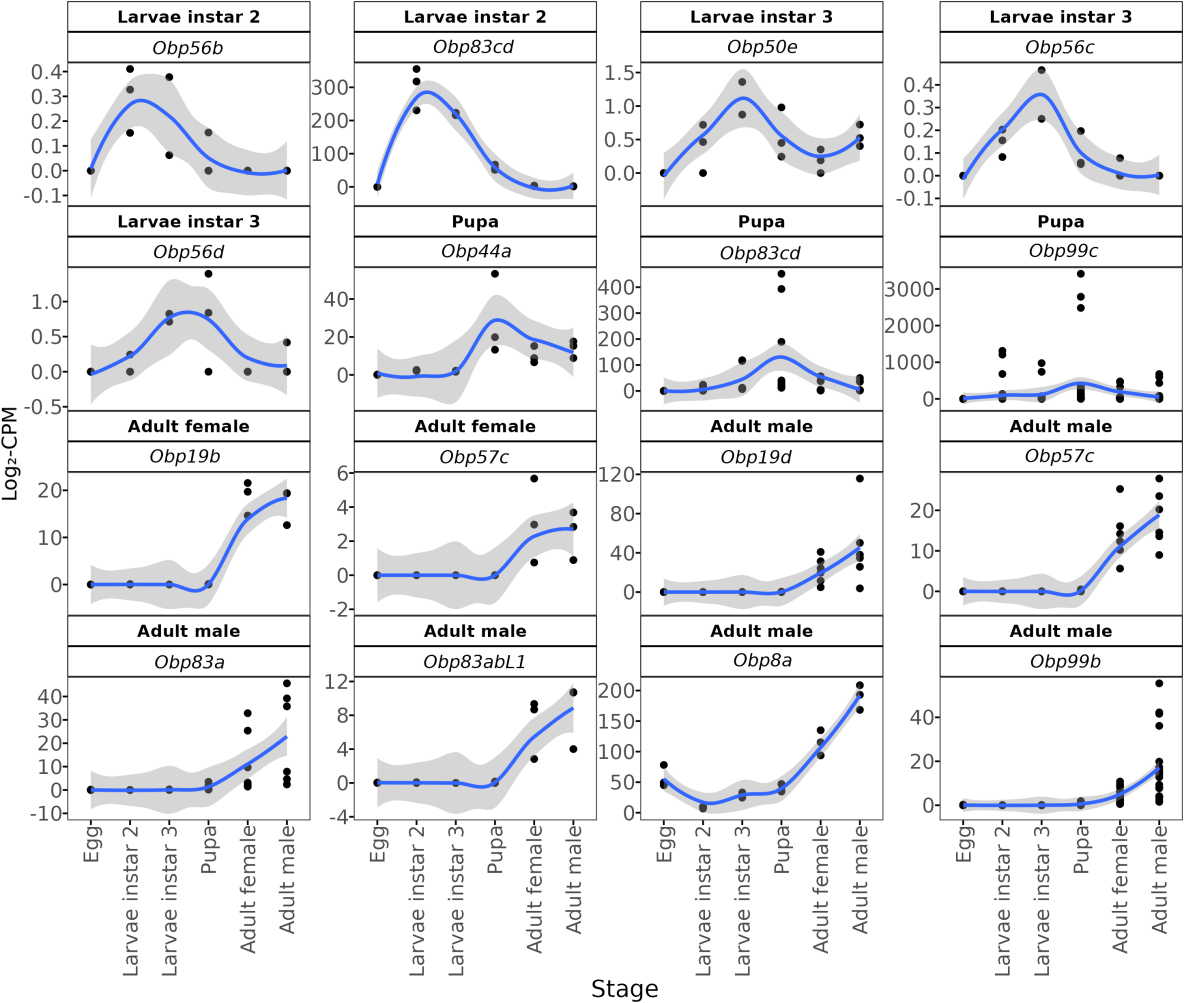
Stage-enriching of DEGs (differentially expressed genes) annotated as odorant-binding protein (OBP). Black dots show log_2_-CPM (counts per million) values for the three biological replicates at each developmental stage (egg, L2, L3, pupa, adult (female and male). The blue line is a LOESS-smoothed curve (gray band, 95% confidence interval) summarizing the expression trend. Panel headers indicate the stage where the transcript reaches its peak expression and its OBP gene or sub-family annotation.

We identified OBPs whose expression profiles show the highest accumulation of transcripts in specific developmental stages (**Figure 4**). *Obp56b* is differentially expressed in the second larval instar and reaches its highest relative transcript levels during this stage, although its overall expression remains low. Similarly, *Obp50e*, *Obp56c*, and *Obp56d* show their highest transcript levels in the third larval instar, relative to other stages, but do not rank among the most highly expressed OBPs overall. Two OBPs (*Obp99c* and *Obp44a*) exhibited a significant gene expression peak during the pupal stage. We identified one OBP (*Obp19b*) with a significant expression peak in adult females. Finally, we found five OBPs transcripts (*Obp8a*, *Obp99b*, *Obp83a*, *Obp83abL1*, and *Obp19d*) significantly expressed in adult males. These findings suggest that different types of OBPs play a critical role in the chemoreception of *A. ludens* during specific developmental stages.

In addition to OBPs, two ionotropic receptors—glutamate receptor ionotropic (or *kainate 2-like*, IDs: UN004236 and UN004237) and sensory neuron membrane protein 2 (ID: UN005667)—as well as the odorant receptor *30a-like* (ID: UN005447), were identified as differentially (or preferentially) expressed genes, the former in third-instar larvae, and the latter two in the pupae.

## 4 Discussion

In this study, we investigated the transcriptional peaks of genes in five developmental stages of the pestiferous tephritid *A. ludens* to gain insight into the specific molecular processes that are active along its development, all the way from eggs to adults. We discovered that the sampled stages clustered in three groups. The eggs were the most distinct, followed by adults, while larvae and pupae were more similar. We identified the most DEGs in the egg stage, with the highest number of peaks related to metabolic pathways. To place our findings on molecular changes along the various developmental stages in an organismal context, we will discuss metabolic DEGs and metabolic pathways, highlighting their physiological significance and roles in the organism’s interaction with the environment.

### 4.1 Cellular communication

The egg stage shows a marked over-representation of transcripts with nucleic-acid-binding functions; most notably, twelve predicted bicoid (*Bcd*) RNA-binding proteins reach their expression maximum in eggs. These proteins bind and anchor *bcd* mRNA to the anterior cortex of the oocyte, establishing the anterior–posterior polarity of the embryo (62). Surprisingly, in the widely distributed pestiferous tephritid *B. dorsalis*, an absence of bicoid proteins has been reported (63).

Cellular communication is of paramount importance in multicellular organisms. The mitogen-associated protein kinase (MAPK) pathway is a crucial link between external signals and intracellular behavior, particularly during embryonic development. Our study revealed a higher expression of unigenes associated with the MAPK signaling pathway in the egg phase (**Figure 3**). The role of this pathway in embryogenesis has been extensively investigated in *D. melanogaster* (64). Moreover, the MAPK pathway is known to be involved in stress responses, including UV radiation, reactive oxygen species (ROS) production, and immune challenges (65).

In *Ceratitis capitata* (Diptera: Tephritidae), the activation of the classical MAPK pathway (ERK 17/2) has been observed in response to peroxide production via the immune response of hemocytes (66). Additionally, it has been demonstrated that both adherent and phagocytic activities of hemocytes in *C. capitata* require activation of this pathway (67). Here, we found that the expression of this pathway is highest during the egg stage, and although it gradually decreases in later stages, its activity remains relatively high compared to basal levels in third-instar larvae, pupae, and adults (**Figure 3**). This suggests that the MAPK pathway may significantly coordinate embryonic developmental events in *A*. *ludens*. Furthermore, the observation of the MAPK as a peaking pathway during the egg stage could indicate the activation of defense mechanisms or the development of immune responses to protect the embryo from potential threats. Interestingly, the second instar larvae did not exhibit the same level of overexpression of many unigenes as observed in the other developmental stages, suggesting a growth-focused phase during this larval feeding stage.

Interestingly, we observed significant enrichment of circadian rhythm-associated genes during the egg stage. In other model insects, such as *D. melanogaster*, although core clock gene transcription begins during embryogenesis, the establishment of a functional circadian system in the central brain is delayed until the second larval instar. The transcriptional activation observed in *A. ludens* eggs may therefore represent an anticipatory expression or reflect activity in peripheral tissues. Nonetheless, our results must be viewed in light of the fact that whole-body RNA extractions were used, which can dilute tissue-specific signals. This is especially relevant for circadian genes, which are known to be highly tissue- and neuron-specific. Thus, further validation using dissected tissues or single-cell RNA-seq would help elucidate the spatiotemporal dynamics of circadian gene regulation in *A. ludens.*

### 4.2 Metamorphosis

The TGF-beta signaling pathway is a crucial regulator of cellular processes such as differentiation, proliferation, migration, and apoptosis, making it essential for tissue homeostasis and development. Extensive research has documented the pivotal role of the TGF-beta pathway in governing tissue and organ differentiation, as well as development during metamorphosis (68). In *D*. *melanogaster*, blocking TGF-beta signaling has resulted in developmental arrest before metamorphosis (69). Similarly, in the hemimetabolous insect *Gryllus bimaculatus* (De Geer) (Orthoptera: Gryllidae), TGF-beta signaling has been implicated as a modulator of metamorphosis through its involvement in the juvenile hormone biosynthesis (70). These findings underscore the conserved role of the TGF-beta pathway in regulating metamorphosis and its intricate interactions with other signaling pathways. In line with these studies, our results show significant overexpression of the TGF-beta pathway during two critical stages of development: the egg and pupal stages.

### 4.3 Growth control and metabolism management

The Forkhead box class O (FoxO) family of transcription factors primarily regulates cell growth and adaptation to nutrient availability. While the transcriptional regulation of FoxO is not well understood, its post-translational regulation has been extensively studied (71). The insulin and/or insulin-like growth factor type 1 (IGF-1) pathway is critical in inhibiting FoxO through phosphorylation. During nutrient scarcity, the insulin/IGF-1 pathway is suppressed, leading to FoxO activation, which promotes the release of glucose and fatty acids as energy sources. Additionally, FoxO inhibits translation and growth, conserving energy for essential survival functions and allowing the organism to adapt to environmental nutrient levels (72). FoxO can also be directly activated or inhibited by extracellular signals, including nutrient levels, cellular stresses (UV and ROS), and developmental signals (73, 74). Overexpression of FoxO in *D*. *melanogaster* has been linked to better responses to various stress stimuli, such as oxidative stress, heat, and immune challenges (75, 76). Notably, silencing FoxO in *D. melanogaster* does not affect its developmental phenotype, except for increased sensitivity to oxidative stress in nutrient-rich conditions (77). Likewise, diets high in sugar inhibit FoxO activation, decreasing lifespan in *D. melanogaster* (78).

Monitoring the expression of FoxO transcripts could offer a practical biomarker for the physiological stress that *A. ludens* experiences under different environmental challenges, including γ-irradiation which is critical for the successful application of the Sterile Insect Technique (SIT). Irradiation has been shown to damage the midgut and alter its resident microbiota (79), impair courtship behavior (80), and reduce adult survival or fecundity unless optimized doses are used (81, 82). In our data, the FoxO signaling pathway peaks first in the egg stage, declines in the second larval instar, and rises again in the third instar, remaining elevated in the adult stage (**Figure 3**). The timing of this second peak coincides with the life stage that is most radio-tolerant in *B. dorsalis* (83), supporting the idea that heightened FoxO activity enhances stress resilience. No genes assigned to the IGF-1 signaling pathway appeared among the stage-enriched DEGs, indicating that their expression remained relatively constant across all stages under our laboratory-rearing conditions. Consequently, FoxO regulation in *A. ludens* is likely governed by a basal IGF-1 input rather than by stage-specific transcriptional surges.

Additionally, the mTOR pathway is also known to regulate FoxO transcription factors. When activated, mTOR suppresses FoxO activity through phosphorylation. Thus, mTOR activity regulates FoxO localization and activity, influencing cellular responses related to metabolism, proliferation, and survival (84, 85). Both mTOR and FoxO are critical cell metabolism and survival regulators, but their activation or inhibition depends on the context and environmental signals. Generally, mTOR activation is associated with nutrient abundance, growth factors, and adequate cellular energy levels, while FoxO activation occurs under nutrient deprivation or cellular stress conditions. The interaction between FoxO and mTOR is crucial for maintaining a proper balance between cell growth and survival in response to environmental conditions and nutrient availability. In this regard, it is important to note that all developmental stages were raised under laboratory conditions with consistent access to a high-quality artificial diet. This environment likely promoted nutrient-rich signaling and mTOR activation, especially during larval stage 2 when growth demands are greatest. The moderate activity of FoxO under these conditions indicates baseline regulatory functions, but its expression patterns may change significantly in field conditions characterized by intermittent food supply or environmental stress. Furthermore, the activity of the FoxO/mTOR signaling pathways in calorie-restricted environments is associated with improved health and longevity in many organisms (86). Exploring these pathways under natural conditions could provide valuable insights into the optimal characteristics for rearing these insects.

The egg stage exhibited the highest number of stage-enriched DEGs in both the mTOR and FoxO pathways, a concordant pattern with their shared roles in metabolic regulation and cell-growth control during early development; however, the latter does not demonstrate direct pathway interaction. This combined expression may help the organism adapt to sudden changes in nutritional resources or environmental challenges. In the second larval instar, the mTOR pathway exhibited a peak of overexpression, contrasting with FOXO. During this developmental stage, the animal may be programmed to cope with optimal food resource conditions. Notably, the mTOR pathway was one of the few pathways overexpressed during the second larval stage and this overexpression of mTOR unigenes persisted until the pupal stage.

Our findings revealed that adult males exhibit a higher number of stage-enriched mTOR-pathway transcripts than females (**Figure 3**). Sex-biased TOR activity has been documented in other dipterans: in *Drosophila*, rapamycin predominantly extends female lifespan via intestinal autophagy (87). Besides, *mTORC1* activity is enriched in male germline stem cells and early spermatogonia, whereas female germline differentiation relies more on insulin/TOR integration (88). Nevertheless, functional assays will be required to determine whether differences in *mTOR* activity contribute directly to sex-specific differences in fitness in this species.

### 4.4 Environmental challenge

The cytochrome P450 family comprises many monooxygenase proteins involved in the oxidation-reduction reactions of various endogenous or xenobiotic compounds. Cytochrome P450 expression is widespread in prokaryotes and eukaryotes, and its functions, in addition to toxin metabolism, include nutrition, growth, and development. This family is highly diverse, to the extent that many isoforms can be found in a single organism, with different tissue expression patterns throughout the life cycle, influenced by environmental factors such as diet (89). Their diversity is such that in *D. melanogaster* alone, 86 genes encoding these proteins have been identified (90), and when comparing among insect species, no more than 30-50% identity has been found. This diversity is observed even within the same order of insects (91).

Endogenous substrates of insect monooxygenases include juvenile hormones and ecdysteroids such as 20-ecdysone and pheromones (92, 93). Targeted activation and/or inhibition of P450s in holometabolous insects results in changes in development, morphology, and survival (94, 95). Therefore, P450 activity is very important for physiological processes such as development. In *A. ludens*, the expression of this protein family peaked during the egg stage, followed by the third larval instar, pupal stage, and adulthood (**Figure 3**). Like most pathways, it was not overexpressed in larval stage 2. The increased expression of cytochrome P450 genes observed in the egg and adult stages may be connected to physiological processes that require higher metabolic activity, like development or detoxification. While it’s tempting to interpret this pattern as an adaptation to environmental exposure—since these stages mark entry and exit points into new environments—this idea is still speculative and needs further research into the specific functions of individual genes.

In instar 2 larvae, which in nature feed within host fruit but in this study developed on an artificial diet, the expression profile is dominated by genes involved in protein synthesis, carbohydrate metabolism and other growth-related processes. Although the nutritional context differs, the relative paucity of differentially expressed genes associated with defense or differentiation, alongside enrichment of growth-related functions, may suggest a physiological focus on somatic development during this stage. However, this pattern should be interpreted with caution as it may also reflect transcriptional continuity from earlier stages or low regulatory turnover. However, upon reaching stage 3, which precedes pupation and metamorphosis, transcription is reactivated, resulting in gene overexpression. In the case of P450, this overexpression could represent preparation for metamorphosis, given its essential role as a hormonal regulator. In contrast to the latter, a study on *B. dorsalis* demonstrated downregulation of P450 genes during the pupation process (96). This contrasting pattern highlights the species-specific regulation of P450 expression during development and underscores the complexity of metabolic adaptations in different insect species; however, alternative explanations like isoform-specific expression or developmental programming cannot be ruled out. Further research is needed to elucidate the specific mechanisms and functional significance of these regulatory patterns in the context of metamorphosis and adaptation to environmental challenges.

### 4.5 Energy metabolism

Carbohydrates, proteins, and lipids are the primary energy sources for living organisms. Lipids play an essential role in the life of insects, as they are not only used for growth and development but also for flight, migration, diapause, starvation, oogenesis, embryonic nutrition, synthesis of sex pheromones, cuticular waxes, and various defensive secretions (97). The variation in nutrient availability faced by organisms determines nutrient intake and metabolism regulation. In this regard, the lack of lipids in an artificial diet used for mass rearing fruit flies reflects the low amount of lipids in the diet of wild flies (*i.e.*, fruit pulp), but this is not a limitation because insect intestinal cells can synthesize fatty acids from sugars or amino acids (98). In addition to the number of nutrients in the diet, the regulation of nutrient metabolism is also due to gene regulation. Among the genes that regulate lipid metabolism are FOXO, ecdysone receptor, and its early genes, sterol regulatory element binding protein (SREBP), hormone receptor-like 96 (*HR96*), hepatocyte nuclear factor (*HR96*), and nuclear factor kappa-light-chain-enhancer of activated B cells (*NF-κB*).

The expression profile of unigenes related to energy metabolism in *A. ludens* highlights the significance of lipid metabolism, which was upregulated across all analyzed stages. Specifically, during the larval stage 2, we recorded a significant overexpression of fatty acids metabolism compared to glucose metabolism (**Figure 3**), suggesting that lipids are used as a primary energy reserve in tephritid flies. In a study investigating the lipid profile throughout the development of *C. capitata*, it was observed that lipids accumulate during the larval phase until the prepupal phase. After that, there is a slight decrease in lipid levels during the pupal phase and the initial stage of the adult phase, followed by a significant decrease toward the final hours of metamorphosis (99). The utilization of lipids for energy production, as reserves, and various processes such as male pheromone synthesis, cuticle formation in both sexes, and egg synthesis in females, requires the upregulation of metabolic machinery dedicated to lipid metabolism.

However, our observations suggest that fatty-acid metabolism in *A. ludens* larvae is directed mainly toward non-energetic functions. Aerobic energy production—indicated by oxidative-phosphorylation transcripts—is low in larval stages but rises markedly in pupae and adults (**Figure 3**). By contrast, anaerobic energy generation relies chiefly on carbohydrates: glycolysis- and gluconeogenesis-related genes are up-regulated at every stage except second-instar larvae (**Figure 3**).

The expression profile of oxidative phosphorylation observed in the different stages of *A. ludens* suggests a preference for anaerobic metabolism in the egg and larval stages. Interestingly, during pupation, *B. dorsalis* exhibits a decrease in the transcription of oxidative phosphorylation genes, further emphasizing the dynamic regulation of metabolic pathways during development (96). Given that energy metabolism is largely regulated by the availability of environmental resources, a more significant activity of anaerobic metabolism could be expected during the natural development of these organisms inside fruits due to the possible lack of oxygen in these; however, in artificial rearing conditions, oxygen is not limited. In this context, it has been reported that *B. dorsalis* increases the expression of genes related to lipid metabolism and gluconeogenesis during anoxic development (100). On the other hand, sensitivity to hypoxia treatments has been demonstrated in tephritid flies, specifically in the egg and larval stages 1 and 2 of *C. capitata* (101). Low expression of mitochondrial respiratory proteins does not necessarily imply a complete inability to use oxygen. Organisms could regulate oxygen utilization more efficiently in these early developmental stages, using limited resources and prioritizing vital functions. Along these lines, a study in *D. melanogaster* showed that mitochondrial oxidative capacity is highly variable during development, but the organism can maintain mitochondrial respiration in all stages (102). Detailed studies of mitochondrial function in tephritid flies are still scarce, rendering aerobic‐energy metabolism a promising target for future research.

### 4.6 Proline/arginine

Proline is the primary precursor for arginine synthesis and vice versa. Arginine and proline metabolism has been shown to protect tephritid and *Drosophila* flies from low temperatures and freezing (103, 104). Furthermore, proline can be an energetic fuel in flying insects, doubling their respiratory capacity (105, 106). Therefore, the expression profile of unigenes involved in this metabolism is of interest, as it may provide clues on their potential role in tephritid flies. Our results show overexpression of these genes in almost all stages studied, except the second larval instar and adult females. Therefore, we do not consider proline an energy source in these flies. Although proline has been linked to cold tolerance in other dipterans, our flies were reared under steady, non-stressful laboratory conditions that do not replicate such environmental pressures. As a result, any proposed role for proline in stress protection remains speculative in this context. Future studies specifically designed to test cold or desiccation stress will be necessary to assess this hypothesis.

### 4.7 Odorant-binding proteins

Like other insects, olfaction in *Anastrepha* species is crucial for locating hosts, mating, foraging, oviposition, and even avoiding predators. There are several strategies for controlling this pest (107), many of them targeting the olfactory system, which comprises seven classes of proteins involved in the olfactory signal transduction pathway (odorant-binding proteins (OBPs), chemosensory proteins (CSPs), odorant receptors (ORs), ionotropic receptors (IRs), odorant degrading enzymes (ODEs), sensory neuron membrane proteins (SNMPs) and Niemann-pick type C2 (NPC2) (108). Of these, OBPs are undoubtedly the best studied. They bind with odorants from the environment and shuttle them to trigger the ORs located on the membranes of olfactory sensory neurons (32, 33).

In the transduction cascade of olfactory signals, the OBPs interact with odorants and transport the chemical signal through the aqueous lymph of sensillas until it reaches the olfactory receptors (32, 33). Its distinct expression pattern, its high molecular divergence and its affinity for specific odorants, suggest that these proteins could act as a filter, selecting the odorants that will trigger the olfactory responses (109). Although not all odorants are necessarily associated with an OBP to stimulate olfactory receptors (110), it has been suggested that a combination of the number and types of OBPs expressed in a species and at which developmental stage and tissues these genes are expressed could influence its specificity and sensitivity to odorants (111, 112).

There are several OBPs expressed mainly in the insect antennas, and it has been proven that many of them are responsible for the detection of pheromones and/or host plant volatiles (113–115); however, it has also been found that OBPs expressed in organs other than the antenna are involved in gustatory perception (109). In *A. ludens*, during its life cycle, *Obp56b* is highly expressed in the larval stages (mainly in the larval stage 2) but not in adults emerging from pupae. It has been reported that orthologs to this protein are expressed in the pharyngeal labral sense organ (LSO) cells in *D. melanogaster*. Pharyngeal LSO belongs to the internal taste organs that help *D. melanogaster* in triggering regurgitation as a response to harmful food or to enhance the sucking/ingestion of appropriate food (116). LSO seems to be stimulated by the presence of sugar, producing a characteristic searching behavior in *D*. *melanogaster* (117). *Anastrepha ludens* larvae are routinely mass-reared on an artificial diet containing ~8% sucrose, a level similar to that found in ripe host fruit pulp (41). Consequently, basal expression, and the early up-regulation, of sugar-responsive OBPs are consistent with larval requirements to detect and ingest carbohydrate-rich food, whether in natural fruit or in the artificial medium provided. OBPs genes such as *Obp56c* and *Obp56d* exhibit their highest transcript levels in the third larval instar. This is consistent with previous studies reporting that ortholog genes in *D. melanogaster* are expressed in all sensilla on the antenna, the maxillary palp, and the larval olfactory system in the dorsal larval organ *(* 117 *)*. It is worth mentioning that other unigenes with similar expression profiles are those homologs coding to the gustatory receptor of trehalose (Trehalose_recep), which can control the feeding response in the presence of sugars (118, 119).

Three OBPs genes with peak expression in the pupal stage have been identified. The ortholog to *obp99c* in *A. obliqua* (Macquart) (which, together with *A. ludens*, belongs to the most derived group within *Anastrepha*) (120) is upregulated in virgin and reproductively active males, suggesting its relevance in mating selection (121). For its part, orthologs to *Obp83cd* in both *A. obliqua* and *A. fraterculus* (Wiedemann), are expressed in immature flies before mating (122). However, OBP99C proteins have also been reported to recognize benzaldehyde in *Drosophila* (123), a volatile compound present in many fruits whose concentration depends on ripeness (124) and is used as a bait component to attract some *Anastrepha* species (125). In a previous study, we reported that the emergence of *Rhagoletis completa* Cresson (Diptera: Tephritidae) adults, a diapausing species, was accelerated in the presence of walnut fruits (126). While there is no evidence of diapause in *A. ludens*, the emergence of adults can be delayed up to several weeks (127). Thus, the up regulation of *Obp99c* may be more critical for *A. ludens* pupae, helping to sense surrounding fruits and synchronize adult emergence. Another OBP identified in the pupa of *A*. *ludens* was *Obp44a*, which has also been reported as upregulated in *B. dorsalis*, playing a role in environmental chemodetection (128).

Some OBPs differentially expressed in adults show distinct expression patterns in males and females. The upregulation of *Obp19b* in females may be related to pheromone recognition and mating. This OBP has also been documented to be upregulated during female maturation in *C. capitata* (129), and in *D. melanogaster*, it is co-modulated with *Obp56h* in mating behavior, which can affect copulation latency (130). Among the five OBPs with peak expression in males, the non-sensory OBP 99b has been used as a marker in *B. dorsalis* to identify male age (131). It is suggested that the function of this OBP, involved in pheromone response or transport, is like that of mammalian lipocalin (132), but further investigation is needed to understand its precise function. However, the upregulation of *Obp99b* in mature *A*. *ludens* males could support the predicted function of this OBP.

We also observed two OBPs transcripts belonging to the *Obp83* cluster (*Obp83a* and *Obp83abL1*) that are mainly expressed in male adults emerging from the pupa. These OBPs play a role in modulating behavioral responses to odorants containing specific functional groups, such as acetophenone and 2-heptanone (133). Acetophenone, a volatile compound found in various plants, can attract or repel certain insect species (134). It specifically attracts females of *Diachasmimorpha longicaudata*, a widespread parasitoid of tephritid flies (135, 136). The last OBP gene with significant expression in males was *Obp19d*. This protein does not exhibit differences between sexes in *D. melanogaster* and is likely involved in detecting food quality. OBP19d proteins are expressed in the proboscis and are present in the olfactory system (137). In a previous study, we observed the upregulation of *Obp19d* in newly emerged and five-day-old antennae of *A*. *ludens* adults exposed to CeraTrap^®^, an attractant formulated with enzymatically hydrolyzed protein obtained from pig intestinal mucosa (138). It is worth noting that the differences between sexes were subtle in this context.

Regarding ionotropic receptors also identified here as differentially expressed, the glutamate receptor (or *kainate 2-like*) is a transmembrane channel activated by glutamate, a neurotransmitter (139, 140). It plays an important role in neural development and central nervous system function (141, 142). Its presence in third-instar larvae developmental stage may suggest that it is critical for the maturation of neural circuits during this period. The upregulation of this receptor could be associated with synaptic plasticity and the formation of neural connections necessary for the larvae to process sensory information effectively, which is crucial as they prepare for the transition to pupation. The sensory neuron membrane protein 2 (SNMP2) identified in the pupae is indicative of the preparation of sensory systems for adult functions. SNMP2 is known to be involved in the detection and processing of pheromones, which are vital for reproductive behavior in adult flies (143–145). Therefore, its increased expression during the pupal stage likely reflects developmental processes required for establishing pheromone detection mechanisms in adults.

The upregulation of the odorant receptor *30a-like* gene during pupation further supports the idea of sensory system refinement in preparation for adulthood. This receptor is part of a broader family responsible for detecting a wide array of odor molecules (146, 147). The differential expression of odorant receptor genes during the pupal stage indicates the reorganization of the olfactory system, ensuring the adult fly will possess a functional and highly responsive olfactory network, critical for locating food, mates, and oviposition sites.

In summary, the differential expression of these ionotropic and odorant receptors underscores the complex molecular transformations that occur during the development of the Mexican fruit fly. These changes ensure that the sensory systems are fully matured and operational by adulthood, allowing the fly to optimally navigate through its environment and fulfill key biological functions.

## 5 Conclusions

Although care was taken to synchronize all sample collections to a common time point (*i.e*., ZT6) during the light phase, we acknowledge that gene expression in many biological processes, including metabolism, signaling, and neuronal activity, may still be modulated by circadian oscillations. If the endogenous clock becomes active earlier than expected in *A. ludens*, some observed differences in gene expression between stages could partly reflect circadian phase rather than the developmental state alone. A more comprehensive temporal sampling strategy across the 24-h cycle would be necessary to fully disentangle these effects. Future studies focusing on high-resolution temporal expression, ideally using tissue-specific sampling, would help clarify the interaction between developmental progression and circadian regulation in this species.

Nonetheless, this study offers an in-depth examination of gene transcription peaks across the five developmental stages of *A. ludens*, providing insight into the specific molecular processes prevalent at each stage. Our findings uncovered significant gene expression patterns that mirror the unique physiological and environmental challenges encountered at each developmental stage. During the egg stage, we identified the greatest number of differentially expressed genes. We observed a notable set of stage-enriched DEGs maps to the Mitogen-Activated Protein Kinase (MAPK) signaling pathway, rendering MAPK one of the prominent stage-peaking pathways, which is well known for relaying extracellular cues to intracellular responses. This intense expression during the egg stage suggests that the MAPK pathway may coordinate significant developmental events in the fly and activate defense mechanisms. Relatively few genes reach their expression peak in the second larval instar, implying that this feeding stage relies mainly on transcripts already active earlier, consistent with a physiological emphasis on continued growth during this feeding phase. However, we observed an overexpression of the Transforming Growth Factor Beta (TGF-beta) pathway, underscoring its conserved role in regulating metamorphosis.

The pupa stage revealed a continued overexpression of the TGF-beta pathway and expression of the mTOR pathway, both crucial for tissue homeostasis and development. Here, we also observed high transcription of the OBP Obp99c, which could play a significant role in mate selection and detecting volatile compounds in fruits. The adult stage displayed distinctive characteristics, with sustained high expression of the FOXO pathway, enhancing stress resistance and crucial for adult survival and reproduction in varied environments. Notably, we observed differences in odor-binding protein (OBP) expression between the sexes. Overexpression of Obp19b in females could be linked to pheromone detection and mating, while Obp99b was overexpressed in males, suggesting a role in pheromone response or transport.

In summary, this study offers a comprehensive view of the distinctive cellular processes across the different developmental stages of *A. ludens*, highlighting the importance of metabolic and signaling pathways and the crucial role of odor-binding proteins. This knowledge could significantly contribute to developing more effective pest management strategies and provide a solid foundation for future research in this critical field. From a practical perspective, our work highlights the need to pay closer attention to egg handling in fruit fly mass-rearing facilities, as our findings show that it is the developmental stage exhibiting the highest number of stage-enriched differentially expressed genes. It is thus likely that if eggs are not adequately handled, the quality of the resulting adults may be compromised.

## Data Availability

The datasets presented in this study can be found in online repositories. The names of the repository/repositories and accession number(s) can be found below: https://www.ncbi.nlm.nih.gov/, PRJNA1253706.
