# Supplementary material for: Stage-resolved transcriptomic profiling of Anastrepha ludens (Diptera: Tephritidae) from egg to adult: molecular signatures of a notorious polyphagous fruit-fly pest
Source: Front Insect Sci. 2025 Aug 29;5:1618382. doi: 10.3389/finsc.2025.1618382 (PMC12426010; doi:10.3389/finsc.2025.1618382)
Supplement: Supplementary file 3 [file Table1.docx]

**Table S1.** Percentage of BUSCO orthologous genes (from the 3,114 Insects reference set) for which transcriptional evidence (CPM > 0) was detected in each developmental stage after independent mapping of RNA-seq libraries to the global *de novo* assembled reference transcriptome.

| **Developmental stage** | **% BUSCO orthologs with CPM > 0** |
| --- | --- |
| Egg | 97.9 |
| Larvae 2 | 98.48 |
| Larvae 3 | 99.02 |
| Pupa | 99.63 |
| Adult female | 99.69 |
| Adult male | 99.59 |

**Note:** These percentages provide an estimate of transcriptomic completeness at each stage, based on detection of BUSCO orthologs using expression thresholds (CPM > 0), and are not derived from *de novo* assemblies per stage.
